# Peer2Me - impact of peer support on self-efficacy in young adult cancer survivors (YA-CS): findings from a comprehensive cohort design

**DOI:** 10.1186/s12885-025-14323-5

**Published:** 2025-05-26

**Authors:** Hannah Brock, Sarah Dwinger, Michael Friedrich, Annekathrin Sender, Kristina Geue, Anja Mehnert-Theuerkauf, Corinna Bergelt, Diana Richter

**Affiliations:** 1 Department of Medical Psychology and Medical Sociology, Comprehensive Cancer Center Central Germany (CCCG), University Medical Center Leipzig, Philipp-Rosenthal-Str. 55, 04103 Leipzig, Germany; 2 Department of Medical Psychology, University Medical Center Hamburg-Eppendorf, Hamburg, Germany; 3 Department of Psychosomatic Medicine and Psychotherapy, Medical Faculty, Otto-von-Guericke-University Magdeburg, Magdeburg, Germany; 4 Department of Medical Psychology, University Medicine Greifswald, Greifswald, Germany

**Keywords:** Young adults, Aya, Cancer, Peer support, Mentoring, Emotional support, Self-efficacy, Psycho-oncology

## Abstract

**Background:**

Numerous studies suggest that young adult cancer survivors (YA-CS) experience unmet needs regarding informational exchange about their disease and emotional support from peers. Currently, there is a lack of evaluated peer support interventions in Germany. This study aimed to evaluate the effectiveness of “Peer2Me”, a three-month one-to-one peer mentoring program, designed to improve self-efficacy among YA-CS.

**Methods:**

This study conducted a bi-center comprehensive cohort design and enrolled YA-CS (18–39 years old) undergoing acute treatment in Leipzig and Hamburg (Germany) across all tumour entities diagnosed within the last six months. YA-CS were allocated by preference to the intervention (IG, receiving peer mentoring) and comparison group (CG, care as usual). Following mentor training, tandems were matched by diagnosis, age, and gender. YA-CS completed questionnaires at baseline (t1), post-intervention (3 months later, t2) and three months post-intervention (t3). The primary outcome was self-efficacy measured with the Generalized Self-Efficacy Scale (GSES) and the Cancer Behaviour Inventory (CBI-B). Statistical analyses included mixed-design ANOVA and ANCOVA, controlling for baseline scores.

**Results:**

Out of 274 eligible YA-CS, a total of 106 YA-CS (IG: *n* = 77, CG: *n* = 29) completed the study. Two-thirds of YA-CS (66.7%) expressed a clear preference to be allocated to the IG. Baseline differences in coping behaviors were noted, with the IG demonstrating lower scores, suggesting a higher need for support. The ANCOVA revealed no group effects for the change of mean GSES scores from t1 to t2 (*p* =.897) or from t1 to t3 (*p* =.779). Also, no significant differences in the improvement of mean CBI-B scores between groups could be found from t1 to t2 (*p* =.903) or from t1 to t3 (*p* =.995).

**Conclusions:**

The “Peer2Me” program did not demonstrate a significant effect on improving self-efficacy among YA-CS during acute treatment. Although interest in peer mentoring was high, the intervention showed no measurable benefit in comparison with standard care. While the need for support was evident, further research is required to optimize peer interventions for this group.

**Trial registration:**

The study was retrospectively registered on February 4, 2022 at clinicaltrials.gov (NCT05336318).

## Background

A diagnosis of cancer in young adulthood represents the experience of a non-normative life event, which gives rise to a distinctive set of physical, social, and psychological challenges. To describe those affected by these challenges, the term “cancer survivor” has also become established, encompassing all individuals currently living with or after a cancer diagnosis [1]. Cancer in adolescents and young adults (AYA), defined by the National Cancer Institute as diagnoses occurring between the ages of 15 and 39, differs significantly from cancer in other age groups due to distinct patterns in cancer types, risk factors, tumor biology, as well as variations in prognosis and survivorship outcomes [2]. Young adult cancer survivors (YA-CS) frequently exhibit elevated levels of psychological distress, including anxiety, depression, and fear of recurrence, as they grapple with the difficulties of a life-threatening disease during a critical developmental period [3, 4]. In contrast to younger patients who may rely significantly on parental support or older adults who may have established family and social structures, the majority of YA-CS reports unmet needs in terms of informational and emotional support [5, 6].

In particular, YA-CS express a desire for social interactions with peers who also had cancer, seeking to share experiences, acquire information, and learn coping strategies that can foster resilience and enhance their self-efficacy [7, 8, 9]. Such interactions with peers can be particularly beneficial in situations where family and friends may feel helpless, thereby limiting their ability to provide the necessary emotional support [10, 11]. Cancer patients often hesitate to share their fears and worries with family and friends, as they do not want to burden their loved ones [10, 11]. Consequently, several studies confirmed that YA-CS want to interact with others of a similar age and diagnosis group [9, 12, 13]. They perceive these relationships as a means of normalizing their experiences, reducing isolation, and obtaining practical advice on managing symptoms and treatments. Interaction with peers on an eye-to-eye level can facilitate identity formation or the development of a positive self-image, providing YA-CS with resources they may not find within their immediate social circles [7, 8, 14, 15]. Nevertheless, in Germany, professional support services that integrate peer support for YA-CS, such as psycho-oncological support or social counseling services, remain scarce. Moreover, there is a paucity of evaluated interventions that utilize peer mentoring as a structured psychosocial support mechanism for this age group.

The evidence for peer support programs demonstrated that interacting with peers who have undergone similar experiences enables YA-CS to benefit from positive coping styles and gain confidence in managing their illness [9, 15, 16]. Heisler et al. emphasizes that peer support is most effective when relationship is non-hierarchical and reciprocal, allowing participants to learn from one another in a supportive and understanding environment [17]. In this context, self-efficacy represents a critical factor influencing the capacity of YA-CS to adapt to and manage the demands of their disease [18, 19]. Defined as the conviction in one’s capability to successfully perform the behaviors necessary to manage prospective situations, self-efficacy influences cancer-related fatigue, health-related quality of life, and psychological adjustment in cancer patients [20, 21, 22]. With regard to YA-CS, low self-efficacy is associated to increased distress, diminished quality of life, and difficulties in engaging in self-care practices and adhering to treatment regimens [23, 24, 25]. Conversely, higher self-efficacy was related to improved psychological outcomes, ability to engage in self-care practices, and a more proactive approach to manage health-related challenges [23, 26]. While evidence supports the value of peer support for YA-CS in terms of enhancing self-efficacy and improving psychosocial outcomes [16, 27, 28], most existing studies focus on group settings or digital interventions [29]. In order to address this gap, we developed the “Peer2Me” intervention with the objective of connecting YA-CS undergoing cancer treatment (mentees) in a 1:1 setting with peers who have already completed their treatment (mentors) over a three-month mentorship period. Peer2Me was previously piloted at Leipzig University Hospital between 2019 and 2020, with successful outcomes regarding feasibility and utilization [30]. The objective of this study was to determine the effectiveness of the Peer2Me intervention in enhancing the self-efficacy of YA-CS (mentees) in the short and medium term. The hypothesis is that participants in the intervention group will report improved self-efficacy compared to participants in the control group after the intervention (t2) and even three months later (t3).

## Methods

### Study design

The study was designed as a bi-center prospective comprehensive cohort study (CCD) [31] with repeated measures to evaluate the effectiveness of the Peer2Me intervention, in which YA-CS were accompanied by a mentor over a period of three months. In this study design, eligible participants in acute treatment could choose to be randomized to the intervention (Peer2Me) or the control condition (a single YA-CS-specific consultation). In addition, patients who were not interested in participating in this randomized trial were asked to complete the accompanying questionnaires (comparison group, standard care). This provided an additional no-intervention comparison group that was relevant for comparison, in particular if the willingness to randomize in the study sample turned out to be very low. Outcomes were measured at baseline before the intervention (t1), immediately after completion of the three-month intervention (t2) and three months after completion of the intervention (t3). Participants received either a link to complete the standardized online questionnaire via Lime Survey, or, if preferred, a printed version of the questionnaire was sent. Detailed information about the study design, recruitment procedure and intervention has already been published in the study protocol [32]. The study received approval from the Ethics Committee of the Faculty of Medicine at the University of Leipzig (Ref. No. 008/21ek) and the University Medical Center Hamburg-Eppendorf (#LPEK-0426).

### Recruitment

YA-CS undergoing acute treatment and mentors were recruited between January 2021 and March 2024 at the cooperating University Medical Centers in Leipzig and Hamburg, Germany. Recruitment was conducted by the study team through the distribution of postal letters or direct personal contact. The invitations to participate in the study were followed up by telephone or email. Information about the study was also provided via study flyers and announcements as well as via social media postings (Facebook, Instagram), thus enabling self-registration. Subsequently, all interested patients completed a contact form and were contacted by the study team to receive detailed study information and to verify inclusion criteria.

Mentees were required to meet the following criteria: (I) cancer diagnosis within the previous 6 months; (II) age between 18 and 39 years; (III) curative treatment approach; (IV) ability to speak fluent German; and (V) written informed consent to participate in this study.

In order to qualify as mentors, individuals had to meet the following criteria: (I) a diagnosis of cancer at least 2 years before study inclusion; (II) current age and age at diagnosis between 18 and 39 years; (III) curative treatment approach; (IV) ability to speak fluent German; (V) written informed consent to participate in this study; and (VI) participation in a mentoring training. To exclude any mental disorders or suicidality, all mentors underwent an interview with a psychologist using the Structured Clinical Interview for DSM-5 Disorders - Clinician Version (SCID-5-CV) prior to the mentor training.

Once acute YA-CS had provided written consent, they were asked to be randomized. Data collection for both mentees and mentors started in January 2022 and was completed in September 2024.

### Intervention

The Peer2Me intervention is a one-to-one mentoring program that aims to provide support to YA-CS undergoing acute treatment by assisting them in effectively coping with the disease [30]. Following the screening process, mentors participated in a mandatory two-day training program, which included key elements of client-centered communication, self-awareness components, disease management as well as a reflection of the own cancer disease and skills related to setting boundaries [32]. Subsequent to the completion of the mentor training program, the tandems were matched according to diagnosis, age, and gender in order to ensure the greatest possible comparability with respect to the cancer disease and treatment [9]. At the inaugural meeting of each tandem, a study staff member elucidated the procedure of the Peer2Me intervention and conducted a baseline assessment (t1). Thereafter, the tandems determined the frequency, duration, and nature of contact (in person or by telephone) over the subsequent three-month period. Following the three-month intervention period, a formal concluding meeting of the tandem was held with a member of the study team, including an evaluation of the contacts and the t2-assessment. The follow-up survey was sent to the mentees three months after the intervention (t3). Group supervision for mentors was provided by a psychotherapist with appropriate expertise in psycho-oncology and group therapy, and this took place once a month for the mentors.

### Outcomes

As outlined in the study protocol, both primary and secondary outcomes were assessed. For the present analysis, we focused on self-efficacy as primary outcome. We used two standardized instruments to assess the extent to which the intervention contributes to an increase in self-efficacy.

#### General self-efficacy scale (GSES)

The German version of the General Self-Efficacy Scale is a validated 10-item scale that is widely used to measure self-efficacy in clinical and non-clinical populations [33]. It measures the expectation of subjective competence to act in the face of challenging situations such as cancer. The items are answered on a four-point Likert scale, ranging from 1 (not at all true) to 4 (exactly true), to determine the extent to which patients agree with the statements. Sum scores range from 10 to 40, with higher scores indicating more optimistic self-beliefs. Normative values for the German general population with an internal consistency of α = 0.92 and a sample of cancer patients with α > 0.90 are available [34, 35].

#### Cancer behavior inventory - brief version (CBI-B)

Self-efficacy in coping with cancer was assessed with the German version of the Cancer Behavior Inventory– Brief Version (CBI-B-D) and can be defined as a cancer patient’s confidence in his or her ability to develop adaptive coping behaviors. Like the original English version, it contains 14 items to describe the individual coping behavior in the context of cancer [36]. In contrast to the original questionnaire, we used a 5-point Likert scale to ascertain the level of confidence respondents exhibited in performing specific behaviors. This scale ranged from 1 (not a tall confident) to 5 (completely confident), instead of a 9-point Likert scale with the objective of enhancing usability and response quality as well as reducing the cognitive burden on patients [37]. Summing the 14 items allows for calculating a total score, ranging from 14 to 70, with higher scores indicating higher confidence in the ability to perform the coping behavior. Four subscale scores represent self-efficacy with respect to (1) Maintaining Independence and Positive Attitude, (2) Participating in Medical Care, (3) Coping and Stress Management, and (4) Managing Affect.

### Statistical analysis

Only participants who completed the self-report questionnaire at all measurement time points were included in the analysis. Mentees who did not use peer support beyond the initial introductory meeting were excluded. Differences in sociodemographic characteristics between completers and drop-outs were assessed using independent samples t-test and Chi-square test of independence. Descriptive analyses were conducted to characterize the sample (total, intervention group, control group) and relevant variables. Baseline differences between the intervention and control groups were tested using independent samples t-tests, with Levene´s test applied to assess equality of variances. To evaluate changes over time, a mixed-design ANOVA with repeated measures was conducted, comparing mean values across three time points (t1, t2, t3) between the intervention and control groups. This analysis assessed main effects of time (within-subject) and group (between-subject). Additionally, analysis of covariance (ANCOVA) was used to assess changes in self-efficacy outcomes between trial arms from baseline to t2 and t3, controlling for baseline scores. Missing outcome data at random were imputed using the expectation-maximization algorithm. All statistical tests were two-tailed, with a significance level of 5%. Analyses were conducted using IBM SPSS Statistics 27.

## Results

### Sample characteristics

**Fig. 1:**
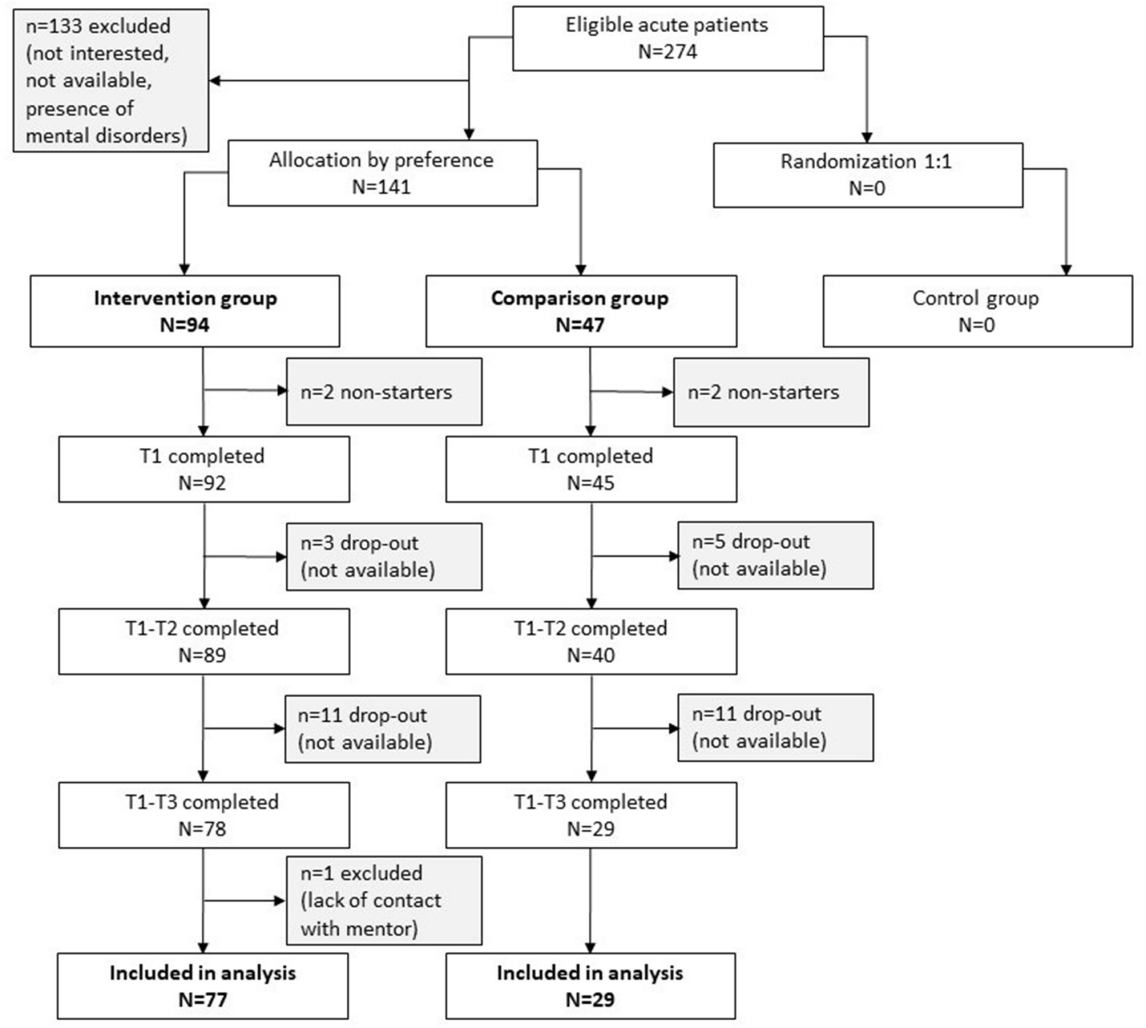
STROBE (Strengthening the Reporting of Observational Studies in Epidemiology) flowchart of participants

Over the 27-month recruitment period, a total of *n* = 274 acute YA-CS met inclusion criteria (Fig. 1). As the CCD allows patient preferences to be taken into account and none of the acute YA-CS agreed to randomization, allocation to the intervention (IG, *n* = 94, 66.7%) and comparison group (CG, *n* = 47, 33.3%) was based on preference and we analyzed data as a two-arm, parallel, nonrandomized prospective cohort study. This resulted in an intervention group consisting of participants who received support from a mentor and a comparison group that received standard care. Due to drop-outs (IG: *n* = 15, 15.9%; CG: *n* = 16, 34.0%), the final sample comprised *n* = 106 participants (IG: *n* = 77, CG: *n* = 29).

Sample characteristics are presented in Table 1. The most prevalent diagnosis was breast cancer (30.2%), followed by hematological (27.4%) and testicular cancer (19.8%). The overall mean age at diagnosis was M = 33.0 years (SD = 5.8). The majority of participants were in a partnership (62.3%) and reported an educational level of more than 10 years (79.2%). No statistically significant differences were observed in sociodemographic and medical characteristics between the IG and CG at baseline (t1). The average time elapsed between the initial cancer diagnosis and the start of the Peer2Me intervention for mentees (IG) was M = 3.5 (SD = 1.8) months.

**Table 1:** Baseline sociodemographic and medical characteristics of the total sample including IG and CG

	*N* (%)				
	Total (*n* = 106)	IG^a^ (*N* = 77)	CG^b^ (*N* = 29)	*p* ^c^	
Age at diagnosis (years), mean (SD)	33.0 (5.8)	32.9 (5.7)	33.5 (6.1)	0.655	
Gender					
Female	73 (68.9)	57 (74.0)	16 (55.2)	0.062	
Male	33 (31.1)	20 (26.0)	13 (44.8)		
Partnership (yes)	66 (62.3)	46 (59.7)	20 (69.0)	0.382	
Children (yes)	40 (37.7)	26 (33.8)	14 (48.3)	1.69	
Education					
≤10 years	22 (20.8)	12 (15.6)	10 (34.4)	0.084	
>10 years	84 (79.2)	65 (84.4)	19 (65.5)		
Currently on sick leave	74 (69.8)	55 (71.4)	19 (65.5)	0.357	
Time since diagnosis (months), mean (SD)	3.5 (1.7)	3.5 (1.7)	3.5 (1.8)	0.995	
Cancer type					
Digestive organs (C15-C26)	2 (1.9)	2 (2.6)	0 (0.0)	0.540	
Bone and articular cartilage	1 (0.9)	0 (0.0)	1 (3.4)		
Skin (C43-C44)	1 (0.9)	1 (1.3)	0 (0.0)		
Mesothelial and soft tissue (C45-C49)	1 (0.9)	1 (1.3)	0 (0.0)		
Breast (C50)	32 (30.2)	27 (35.1)	5 (17.2)		
Female genital organs (C51-C58)	9 (8.5)	7 (9.1)	2 (6.9)		
Male genital organs (C60-C63)	21 (19.8)	12 (15.6)	9 (31.0)		
Thyroid, endocrine glands (C73-C75)	10 (9.4)	6 (7.8)	4 (13.8)		
Hematological (C81-C96)	29 (27.4)	21 (27.3)	8 (27.5)		
Treatment^d^					
Chemotherapy	60 (56.6)	46 (59.7)	14 (48.2)	0.640	
Radiotherapy	26 (24.5)	23 (29.9)	3 (10.3)	0.343	
Surgery	78 (73.6)	60 (77.9)	18 (62.0)	0.567	
Stem cell transplant	4 (3.8)	3 (3.9)	1 (3.4)	0.481	

Notes. Abbreviations: M = mean, SD = standard deviation, IG = intervention group, CG = comparison group

^a^ Intervention group = Mentees who participated in the Peer2Me intervention

^b^ Comparison group = Care as Usual

^c^*p* type-I-error probability

^d^ Multiple answers possible, cases do not add up to the total number

### Drop-out analysis and missing values

The analysis revealed significant differences between completers and drop-outs concerning education level at baseline. Among drop-outs, 38.7% (*n* = 12) had an education level of ≤ 10 years, while 61.3% (*n* = 19) had > 10 years of education. In contrast, among completers, 20.8% (*n* = 22) had an education level of ≤ 10 years, and 79.2% (*n* = 84) had > 10 years of education, χ²(1) = 4.144, *p* =.042, φ = 0.042. No differences were observed between drop-outs and completers regarding age at diagnosis, time since diagnosis, gender, partnership status, children, cancer type and treatment.

Regarding the primary outcome measures, missing data were observed on the GSES for 8.5% (*n* = 9) of participants. For the CBI-B, missing values were imputed for 25.5% (*n* = 27) of the sample.

### Self-efficacy

**Fig. 2:**
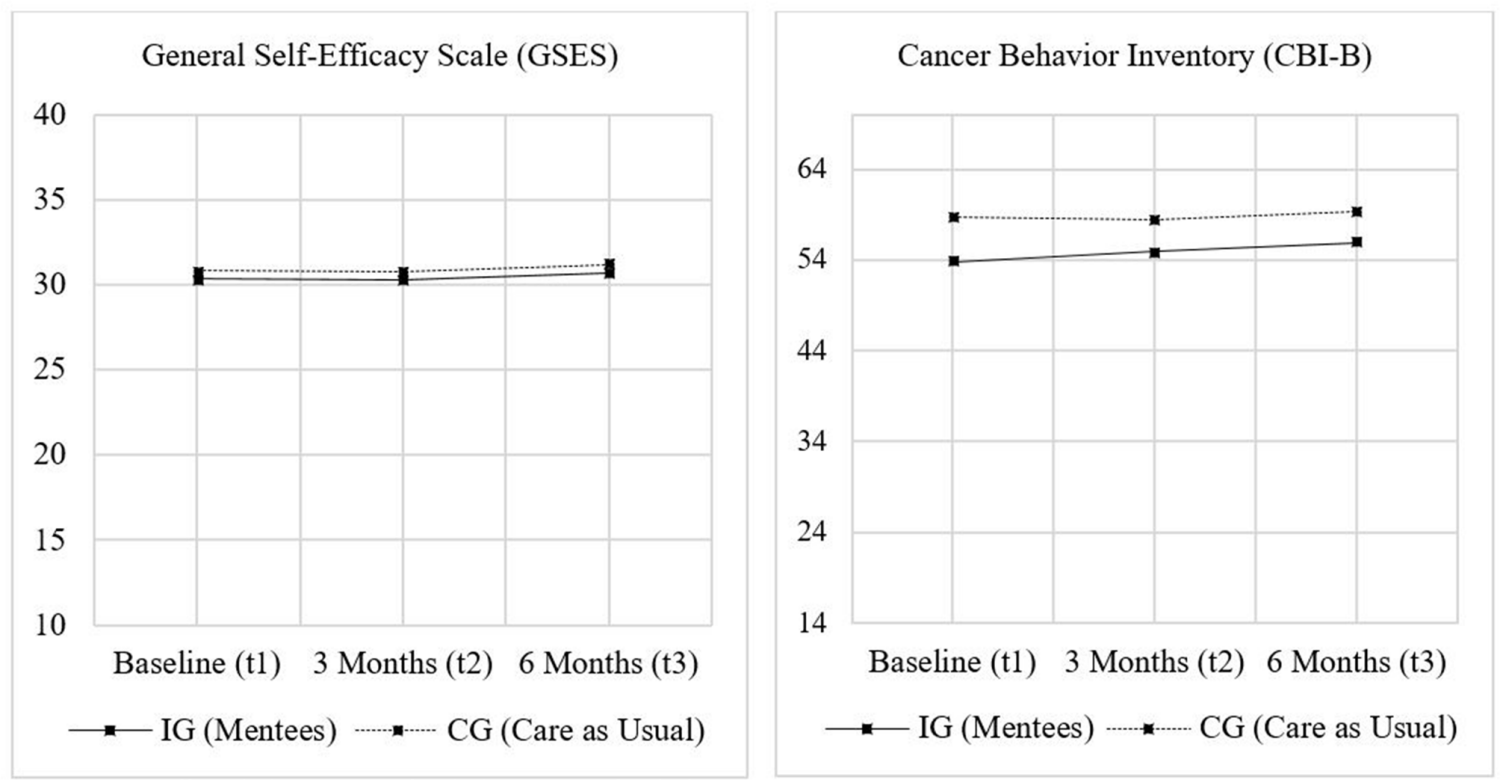
Course of the mean General Self-Efficacy Scale (GSES) scores and the mean Cancer Behavior Inventory (CBI-B) scores for IG and CG

A mixed ANOVA revealed no statistically significant time x group interaction (F(2, 202) = 0.003, *p* =.997, ηp²<0.001) and no significant main effects of time (F(2, 202) = 0.800, *p* =.451, ηp²=0.008) or group (F(1, 101) = 4198.452, *p* =.604, ηp²=0.003), on mean GSES scores. Similarly, ANCOVA analyses, controlling for baseline scores, showed no group effects for the change of mean GSES scores from t1 to t2 (F(1, 102) = 0.017, *p* =.897, ηp²<0.001) or from t1 to t3 (F(1, 100) = 0.079, *p* =.779, ηp²=0.001). Figure 2 illustrates the course of mean GSES and CBI-B scores for the IG and CG.

Regarding mean CBI-B scores, the mixed ANOVA with a Greenhouse-Geisser correction indicated no significant interaction between time and group (F(1.82, 187.76) = 0.589, *p* =.541, ηp²=0.006) and no significant differences across measurements (F(1.82, 187.76) = 1.68,*p* =.191, ηp²=0.016). However, a significant group effect was found (F(1, 103) = 5.273, *p* =.024, ηp²=0.049), yet post-hoc analysis revealed that the only significant difference in mean CBI-B scores between IG and CG was observed at baseline (*p* =.010). ANCOVA analyses (controlling for baseline scores) revealed no significant differences in the improvement of mean CBI-B scores between IG and CG, neither from t1 to t2 (F(1, 103) = 0.015, *p* =.903, ηp²<0.001) nor from t1 to t3 (F(1, 102) < 0.001, *p* =.995, ηp²<0.001). Table 2 provides a concise summary of the GSES and CBI-B mean sum and subscale scores at baseline as well as group differences regarding the changes from baseline to 3 (t2) and 6 months (t3) presented in the preceding section.

**Table 2:** Mean Scores for GSES and CBI-B among the intervention (IG) and comparison group (CG)

	Baseline, Mean (SD)			3 months change, group difference			6 months change, group difference		
Psychosocial Outcome	IG	CG	*p* ^*a*^	IG	CG	*p* ^*b*^	IG	CG	*p* ^*b*^
General Self-Efficacy Scale (GSES)	30.3 (4.9)	30.8 (5.1)	0.631	−.01 (3.41)	−.07 (3.24)	0.897	.35 (3.39)	.38 (3.47)	0.779
Cancer Behavior Inventory (CBI-B)	53.8 (8.6)	58.7 (8.7)	.010*	1.13 (7.21)	−.24 (6.24)	0.903	2.09 (8.04)	.62 (6.95)	0.995
Maintaining Independence and Positive	4.2 (.7)	4.4 (.7)	0.122	.03 (.67)	.02 (.68)	0.519	.17 (.65)	.07 (.62)	0.941
Participating in Medical Care	3.9 (.7)	4.2 (.7)	.018*	.07 (.67)	−.07 (.47)	0.952	.09 (.75)	−.03 (.61)	0.892
Coping and Stress Management	3.4 (.8)	4.0 (.9)	.001**	.20 (.77)	.01 (.78)	0.827	.26 (.80)	.09 (.72)	0.578
Managing Affect	3.9 (.9)	4.9 (1.1)	0.488	.01 (.80)	.07 (.85)	0.355	.07 (.86)	.10 (.76)	0.512

Notes. Abbreviations: SD = standard deviation, IG = intervention group, CG = comparison group, *p* = type-I-error probability. Bold type values indicate statistical significance

^a^*p*-Values based on independent samples t-test

^b^*p*-Values based on analysis of covariance (ANCOVA) controlling for baseline scores, between group differences, values at each follow up compared with baseline

*p <.05

**p <.005

## Discussion

The objective of this study was to evaluate the effectiveness of the Peer2Me intervention in enhancing the self-efficacy among YA-CS. Regarding the drop-out analysis, it was found that a significant higher proportion of YA-CS with a higher level of education (> 10 years) completed the study compared to those who dropped out.

The primary finding revealed no significant changes in self-efficacy (primary outcome), either within groups over time, or between the IG and CG as measured by the GSES, even when controlling for baseline differences between groups. In addition, at the 3-month follow-up, the IG demonstrated a slight improvement on the CBI-B total score and two subscales (Participating in Medical Care, Coping and Stress Management). However, these differences in changes from baseline to 3 and 6 months were not statistically significant when compared to the CG. Thus, participation in the Peer2Me intervention (provision of social support from a mentor) did not lead to a significant improvement in self-efficacy or coping mechanisms compared to standard care in our study sample.

Comparing the findings with other peer support studies remains challenging due to the variability in design, including the format, duration and frequency of the intervention as well as assessment instruments used [38]. Nonetheless, our results are in contrast with our pilot study and prior empirical studies that reported positive effects of peer support programs on self-efficacy and other psychosocial outcomes among YA-CS and older cancer patients [16, 27, 28, 39]. A systematic review identified five studies demonstrating small to moderate effects of peer support on improving self-efficacy in cancer patients. However, these studies primarily focused on older patients with prostate cancer, and self-efficacy was assessed using different measurement instruments [28]. The research group led by Casillas and colleagues [27] examined the impact of peer navigation interventions compared to standard care among YA-CS. Their findings indicated that peer support was more effective than standard care in enhancing self-efficacy. The intervention was based on reading an educational YA-CS booklet and deriving individual goals, which were reviewed through two phone calls with a peer, which differs from the approach used in our study design. Their sample also consisted of childhood cancer survivors, who were examined on average eight years after completing treatment.

In contrast, Toija et al. reported that peer support had no effect on health-related quality of life. They investigated the impact of peer support delivered via telephone (one to five calls) exclusively among (older) breast cancer patients. The authors attributed these findings to the low intensity of the intervention and the lack of control over the use of external peer support in the control group [40]. The mixed results observed across studies can largely be attributed to the heterogeneity in the design and delivery of peer support interventions, as well as differences in sample characteristics.

The current literature confirms that peer support is most efficacious when implemented at an early stage and over a longer period of time [38, 41]. This indicates that a three-month intervention may be too short to produce measurable effects on psychosocial variables, such as self-efficacy. As observed by Giese-Davis et al. [42], a six-month peer-counseling intervention for women newly diagnosed with breast cancer resulted in effects on quality of life that were discernible over a period of 12 months. In principle, the duration of the peer support intervention varies in previous studies from 4 weeks to 12 months [28]. Furthermore, the mean time since diagnosis in our sample exceeded three months, which may have been too late to observe meaningful changes. This could have also reduced the impact of peer support, as mental distress typically decreases over time following a cancer diagnosis [41].

Another potential explanation for the absence of changes over time and between groups is that self-efficacy represents a particularly stable construct, making it challenging to demonstrate significant changes over relatively short periods of time [43, 44]. It should also be noted that self-efficacy may not fully capture all of the factors that are influenced by peer support (e.g. anxiety, depression, distress etc.) [28, 38].

Another key finding of the study concerning the recruitment process is that none of the YA-CS agreed for randomization, instead making a deliberate decision regarding group allocation. Consequently, their preferences were taken into account in accordance with the study design. A clear preference for the IG was expressed by two-thirds of YA-CS, which emphasizes the necessity of peer support in the context of acute cancer treatment. The analysis of baseline values demonstrated that the IG exhibited significantly lower levels of self-efficacy and coping behaviour. This outcome suggests that YA-CS with less robust coping mechanisms may have a greater need for social support and mentorship in navigating their illness and treatment. This finding is corroborated by numerous empirical studies [45, 46]. Although baseline values were controlled in the ANCOVA, it cannot be ruled out that the IG was more burdened compared to the CG due to less pronounced coping strategies. This finding suggests that the effects of the Peer2Me intervention may not have fully manifested within the observed timeframe.

### Strengths and limitations

This study is the first to evaluate a structured peer mentoring program for YA-CS in Germany, marking an innovative approach. The comprehensive cohort design allows for a differentiated investigation of psychosocial outcomes, with a special focus on patient preferences. Using validated and standardized instruments for self-efficacy enhances the study’s methodological rigour. In addition, the triple data collection across different time points provides valuable insights into the dynamics of the intervention. A further strength of the study is the demographic comparability of the IG and CG, ensuring that differences in sample characteristics do not confound the results.

A major limitation of the study is the absence of control over the nature and intensity of the mentoring relationship. In our study, the frequency of peer support interactions was largely determined by patient preferences, with some YA-CS requiring only a few phone calls, while others preferred weekly in-person meetings. Unfortunately, we were unable to systematically document the exact number and nature of contacts for each patient. This variability in the practical implementation of the mentoring relationship, including factors such as type, frequency, and intensity of contact, is a crucial element that could have influenced outcomes. However, due to the lack of systematic recording and control over these variables, their potential impact remains unclear.

Furthermore, the results may have been influenced by a ceiling effect, as both the IG and the CG demonstrated scores within the upper range of the analyzed instruments at all measurement time points. It is therefore also necessary to consider the possibility of a selection bias, given that the composition of the sample may have been influenced by the conditions of participation. In particular, patients who were undergoing multimodal intensive therapies that had to be carried out exclusively and over a longer period of time as inpatients (e.g. sarcomas or hematological malignancies) may have encountered difficulties in participating due to logistical challenges [2, 47]. This may have resulted in a sample comprising YA-CS with less invasive treatment settings and lower need for support. However, the observed selection bias may also be attributed to the overrepresentation of YA-CS with higher levels of education in our sample, as this is often associated with stronger self-efficacy and the utilization of functional coping mechanisms in cancer survivors [48]. The higher proportion of drop-outs with lower education levels and their underrepresentation in the sample serve to reinforce this hypothesis.

Moreover, the study was conducted in two large university hospitals, and it cannot be ruled out that the specific care structures in these settings may have made it more difficult to detect group differences between the IG and CG. In such facilities, care is typically more comprehensive and centered around specialized multidisciplinary teams. As these teams already provide intensive support, the need for, or impact of additional peer support may therefore be reduced. Furthermore, the sample size and the heterogeneous composition of the participants in terms of their diagnoses and treatment phases restrict the extent to which the results can be generalized. The smaller sample size compared to the target specified in the study protocol can be attributed to the recruitment taking place during the COVID-19 pandemic and the fact that postal contact with patients was only permitted at the Leipzig site.

### Research and clinical implications

Future studies should include larger samples and longer intervention periods as well as greater control of heterogeneity in practical implementation so that more robust conclusions can be drawn. It would be highly valuable to investigate the effects of cancer peer support programs in YA-CS in greater detail. For instance, future studies could directly compare different components or formats of peer support, such as variations in modality or duration [39].

In addition, it may be beneficial to include YA-CS at the earliest possible stage following diagnosis into the study, in order to facilitate the analysis of the effects of peer mentoring during this particularly vulnerable period.

Given the underrepresentation of YA-CS from lower educational backgrounds in clinical studies and the association between education level and self-efficacy, the effectiveness of peer support should also be investigated among YA-CS with lower educational levels. Temporal or financial barriers may present challenges to study participation, and the potential benefits of peer support could be particularly pronounced in this group.

In light of the observed allocation preference in the sample, it is crucial to note that the absence of a group effect does not necessarily diminish the clinical relevance of the Peer2Me intervention or peer support programs in general. In this context, an analysis of the qualitative data provided by YA-CS may indicate individual improvements and subjective benefits. We are currently examining these benefits based on interviews with the participants who were offered peer support (IG) and we will report the results in a subsequent article.

In order to optimize the effectiveness of peer interventions in a clinical setting, it is recommended that they be customized and adapted in a manner that is more closely aligned with the specific needs of YA-CS. For instance, closer support for the tandems would be a beneficial addition.

## Conclusions

Despite the absence of significant differences between the IG and CG in terms of self-efficacy change over time, the study provides valuable insights into the need, feasibility and acceptability of the Peer2Me intervention. The findings indicate that peer mentoring could be a promising approach for YA-CS, but it requires further optimization and rigorous evaluation. The methodological challenges and limitations identified in this study highlight areas for the further refinement in the design and implementation of peer support programs. In the long term, peer support could become an integral component of the psycho-oncological care provided to YA-CS. To fully unlock the potential benefits of such programs, further research and greater standardization are necessary. Developing personalized and evidence-based peer support models tailored to the unique needs of YA-CS remains an important task for future research efforts.

## Data Availability

The datasets generated and analyzed during the current study are available from the corresponding author on reasonable request.

## Abbreviations

AYA: Adolescents and young adults
YA-CS: Young adult cancer survivors
CCD: Comprehensive cohort design
IG: Intervention group
CG: Comparison group
GSES: General self-efficacy scale
CBI-B: Cancer behaviour inventory– brief version
